# Urinary protein-to-creatinine ratio in pregnant women after dipstick testing: prospective observational study

**DOI:** 10.1186/s12884-015-0776-9

**Published:** 2015-12-14

**Authors:** Yosuke Baba, Takahiro Yamada, Mana Obata-Yasuoka, Shun Yasuda, Yasumasa Ohno, Kosuke Kawabata, Shiori Minakawa, Chihiro Hirai, Hideto Kusaka, Nao Murabayashi, Yusuke Inde, Michikazu Nagura, Hiromi Hamada, Atsuo Itakura, Akihide Ohkuchi, Makoto Maeda, Norimasa Sagawa, Akihito Nakai, Soromon Kataoka, Keiya Fujimori, Yoshiki Kudo, Tomoaki Ikeda, Hisanori Minakami

**Affiliations:** Departments of Obstetrics and Gynecology, Jichi Medical University Hospital, Shimotsuke, Japan; Department of Obstetrics, Hokkaido University Graduate School of Medicine, Kita-ku N15 W7, Sapporo, 060-8638 Japan; Departments of Obstetrics and Gynecology, University of Tsukuba Hospital, Tsukuba, Japan; Departments of Obstetrics and Gynecology, Fukushima Medical University Hospital, Fukushima, Japan; Departments of Obstetrics and Gynecology, Ohno Ladies Clinic, Iwakura, Japan; Departments of Obstetrics and Gynecology, Hakodate Central General Hospital, Hakodate, Japan; Departments of Obstetrics and Gynecology, Hiroshima University Hospital, Hiroshima, Japan; Departments of Obstetrics and Gynecology, Juntendo University Hospital, Tokyo, Japan; Departments of Obstetrics and Gynecology, Mie Chuo Medical Center, Tsu, Japan; Departments of Obstetrics and Gynecology, Mie University Hospital, Tsu, Japan; Departments of Obstetrics and Gynecology, Nippon Medical School Tama Nagayama Hospital, Tama, Japan; Departments of Obstetrics and Gynecology, Rakuwakai Otowa Hospital, Kyoto, Japan

**Keywords:** Gestational hypertension, Kidney function, Preeclampsia prediction, Protein creatinine ratio, Proteinuria pregnancy, Urine dipstick

## Abstract

**Background:**

The dipstick test is widely used as a primary screening test for detection of significant proteinuria in pregnancy (SPIP). However, it often shows a false positive test result. This study was performed to determine which pregnant women should be recommended to undergo determination of urinary protein-to-creatinine ratio (mg/mg, P/Cr test) after dipstick test for confirmation of SPIP.

**Methods:**

This was a multicenter, prospective, and observational study of 2212 urine specimens from 1033 pregnant women who underwent simultaneous dipstick and P/Cr tests in the same spot urine samples at least once. SPIP was defined as P/Cr > 0.27. Preeclampsia was diagnosed in women with both hypertension and SPIP.

**Results:**

Preeclampsia, hypertension alone, and SPIP alone developed in 202 (20 %), 73 (7.1 %), and 120 (12 %) women, respectively. Creatinine concentration [Cr] varied greatly, ranging from 8.1 to 831 mg/dL in the 2212 urine samples. Rate of positive dipstick test results increased with increasing [Cr], while SPIP prevalence rate was lower in urine samples with higher [Cr], yielding higher false positive rates in samples with higher [Cr]. Postpartum urine samples had significantly lower [Cr] compared to those obtained antepartum (60 [8.7–297] vs. 100 [10–401] mg/dL, respectively). At the first P/Cr test among women with similar dipstick test results, the risk of having SPIP was consistently and significantly higher for hypertensive women than for normotensive women at any dipstick test result: 18 % (14/77) vs. 3.2 % (8/251), 47 % (26/55) vs. 8.7 % (37/425), 91 % (82/90) vs. 59 % (44/75) for negative/equivocal, 1+, and ≥ 2+ test results, respectively. The risk of SPIP was 16 % (9/55) for normotensive women when two successive antenatal urine samples showed a dipstick test result of 1 + .

**Conclusions:**

For prediction of SPIP, the dipstick test was more likely to show a false positive result in concentrated urine samples with higher [Cr]. Hypertensive women with ≥ 1+ as well as normotensive women with ≥ 2+ on dipstick test should be advised to undergo the P/Cr test.

## Background

Preeclampsia (PE) is a life-threatening complication for both the mother and fetus [1]. As PE is usually diagnosed in women that developed both hypertension and significant proteinuria in pregnancy (SPIP), assessment of proteinuria is an important constituent of antenatal care for pregnant women. The dipstick test is widely used in screening for SPIP, but concerns have been raised regarding its accuracy [2–8]. Both false negative and positive results can occur on dipstick test for prediction of SPIP [2–10]. The dipstick test is designed to reflect urinary protein concentration [P] at a certain cut-off level not corrected by urinary creatinine concentration [Cr]; dipsticks with ≥ 1+ on visual judgment for urinary protein concentration ≥ 30 mg/dL are widely used in Japan [9]. It has been suggested that negative and positive dipstick test results are likely to occur in diluted and concentrated urine samples with lower and higher creatinine concentrations, respectively, regardless of actual daily protein loss in the urine [9].

The golden standard test for determination of SPIP is confirmation of urinary protein loss ≥ 0.3 g/day in urine collected for 24 h (24-h urine test). However, the 24-h urine test is inconvenient for both pregnant women and obstetric service providers, and it is sometimes difficult for pregnant women to collect 24-h urine accurately [9, 11]. As daily creatinine production reflects muscle mass and creatinine is eliminated solely by renal excretion, 24-h urinary creatinine excretion reflects muscle mass and excretion is relatively constant over time in a given person [12], ranging from 11.0 mg/kg/day to 25.0 mg/kg/day [11]. Use of the urinary spot protein-to-creatinine ratio (P/Cr test) is currently recommended for evaluation of protein loss per day outside pregnancy [13, 14]. The Australian Society for the Study of Hypertension in Pregnancy, the International Society for the Study of Hypertension in Pregnancy, and the Japan Society of Obstetrics and Gynecology have proposed use of the P/Cr test as an alternative to 24-h urine collection [15–17], and a threshold of 30 mg/mmol (0.265 mg/mg) is recommended [15–18]. In Japan, confirmation by P/Cr test is recommended in pregnant women with dipstick test results ≥ 1+ in the presence of hypertension, ≥1+ on two successive antenatal care visits, and ≥ 2+ even in the absence of hypertension [17]. However, the rationale for this recommendation has not been examined in detail. Thus, it is unclear which pregnant women should be recommended to undergo confirmation of SPIP with P/Cr test after obtaining positive dipstick test results.

This multicenter prospective observational study was conducted to determine the predictive capability of the dipstick test for SPIP in pregnant women in various clinical situations and which pregnant women should be recommended to undergo determination of urinary protein-to-creatinine ratio (mg/mg, P/Cr test) after dipstick test for confirmation of SPIP.

## Methods

This prospective observational study was conducted after receiving approval from the Institutional Review Board of Hokkaido University Hospital (013–0399, April 30, 2014). The following 12 facilities located throughout Japan participated in this study: Hiroshima University Hospital (HUH, Hiroshima), Rakuwakai Otowa Hospital (ROH, Kyoto), Ohno Ladies Clinic (OLC, Nagoya), Mie Chuo Medical Center (MCMC, Tsu), Mie University Hospital (MUH, Tsu), Nippon Medical School Tama Nagayama Hospital (NMSTNH, Tokyo), Juntendo University Hospital (JUH, Tokyo), University of Tsukuba Hospital (UTH, Tsukuba), Jichi Medical University Hospital (JMUH, Shimotsuke), Fukushima Medical University Hospital (FMUH, Fukushima), Hakodate Central General Hospital (HCGH, Hakodate), and Hokkaido University Hospital (HoUH, Sapporo).

### Participants

A total of 6984 women gave birth at gestational week (GW) ≥ 22 during the one-year study period between April 1, 2014, and March 31, 2015, in the 12 facilities. Of the 6984 women, 1033 (15 %) underwent simultaneous dipstick test and P/Cr test in the same spot urine specimens at least once, and were enrolled in this study. All 1033 participants gave verbal informed consent to the participation in this study after opening details of this study to the public via the website of Hokkaido University Hospital in June 2014.

Various information was collected regarding these 1033 women, i.e., maternal age, experience of prior birth, gestational week (GW) at simultaneous dipstick and P/Cr tests with results of those tests, development of hypertension, GW at delivery, delivery mode, infant birthweight, and reasons for the first performance of P/Cr test that were classified into the following seven categories according to dipstick test results and the presence or absence of hypertension: (1) negative or equivocal dipstick test results in the presence of hypertension, (2) positive dipstick test result (1+) in the presence of hypertension, (3) positive dipstick test result (≥2+) in the presence of hypertension, (4) negative or equivocal dipstick test results in the absence of hypertension, (5) positive dipstick test result (1+) in the absence of hypertension, (6) positive dipstick test result (≥2+) in the absence of hypertension, (7) positive dipstick test result (1+) on two successive antenatal care visits in the absence of hypertension.

SPIP was defined as a P/Cr (mg/mg) test result of > 0.27. Hypertension was defined as the occurrence of SBP ≥ 140 mmHg and/or DBP ≥ 90 mmHg. PE was diagnosed in women that showed both hypertension and SPIP. PE was also diagnosed in women with chronic hypertension when SPIP occurred on and after GW 20. Gestational hypertension was defined as a new onset of hypertension on and after GW 20 in the absence of SPIP throughout gestation. Chronic hypertension was diagnosed in women who were confirmed to have hypertension before GW 20.

### Dipstick test and P/Cr test

Spot urine sampling was not performed at any particular time of the day, and without any particular time in relation to meal intake for outpatients. Morning urine was served for the dipstick and P/Cr tests for inpatients. The dipstick examination to screen for SPIP was performed by nurses or midwives. Various types of dipstick were used, including Combisticks™, Uristicks™, N-Multisticks SG-L™, Hemacombisticks™, and Lovesticks™ (Siemens Healthcare Japan Co., Ltd., Tokyo) at nine facilities (JUH, FMUH, HCGH, OLC, NMTNH, HUH, UTH, MUH and HoUH), Uropaper III™ (Eiken Chemical Co., Ltd., Tokyo) at two facilities (JMUH and ROH), and Meditape II 9U™ (Arkray Inc., Kohga, Japan) at one facility (MCMC). Most dipsticks used in this study are likely to show a false positive test result in urines with high specific gravity, urines of alkaline with pH ≥ 8.0, and or urines contaminated with quaternary ammonium salt or antiseptic such as a chlorhexidine, and a false negative result in acidic urines with pH ≤3.0 according to manufacturer's instructions. The attending physicians at each facility ordered the P/Cr test in the same spot urine specimen with a dipstick test result at their discretion when it was considered helpful for patient care. Measurement of protein concentration ([P], mg/dL) and [Cr] were performed at the institutional central laboratory of each of the 12 participating facilities.

Data are presented as the median (range). Statistical analyses were performed using the JMP10© statistical software package (SAS, Cary, NC). The Wilcoxon/Kruskal–Wallis method was used for comparison of medians. Fisher’s exact probability test was used for comparison of categorical variables. In all analyses, *P* < 0.05 was taken to indicate statistical significance.

## Results

SPIP was confirmed in 322 of the 1033 women (31.2 %) enrolled in the study. Of the 322 women with SPIP, 202 had hypertension and were diagnosed with PE, while the remaining 120 women were diagnosed as having SPIP alone (Table 1). Seventy-three (7.1 %) women developed hypertension alone and were diagnosed with gestational hypertension. A total of 2212 simultaneous dipstick and P/Cr tests were performed antepartum and/or postpartum in the 1033 women (Table 1); only once in 402 women, twice in 207, three times in 339, four times in 46, five times in 39 women, only antepartum in 826 women, only postpartum in 29 women, and both ante- and postpartum in 178 women.

**Table 1:** Demographic characteristics of 1033 participants

Number of study subjects	1033
Maternal age (yr)	33 (16–49)
Nulliparity	583 (56.4 %)
Twin pregnancy	95 (9.2 %)
Gestational hypertension	73 (7.1 %)
SPIP alone	120 (11.6 %)
Preeclampsia	202 (19.6 %)
Gestational week at delivery for singletons	38.4 (23.7–42.3)
Birthweight for singletons (g)	2872 (369–4645)
Gestational week at delivery for twins	37 (24.3–40)
Birthweight for twins (g)	2282 (125–3276)
Caesarean delivery for singletons	433 (46.1 %)
Caesarean delivery for twins	82 (97.6 %)
Total number of P/Cr tests^a^	2212
Number of P/Cr tests/person	2 (1–5)
Antenatal P/Cr test^a^ alone	826 (79.9 %)
Postnatal P/Cr test^a^ alone	29 (2.8 %)
Both of the above	178 (17.2 %)

Data are presented as the median (range) or number (percentage)

SPIP, significant proteinuria in pregnancy defined as P/Cr > 0.27;

^a^with simultaneous dipstick test

**Fig. 1:**
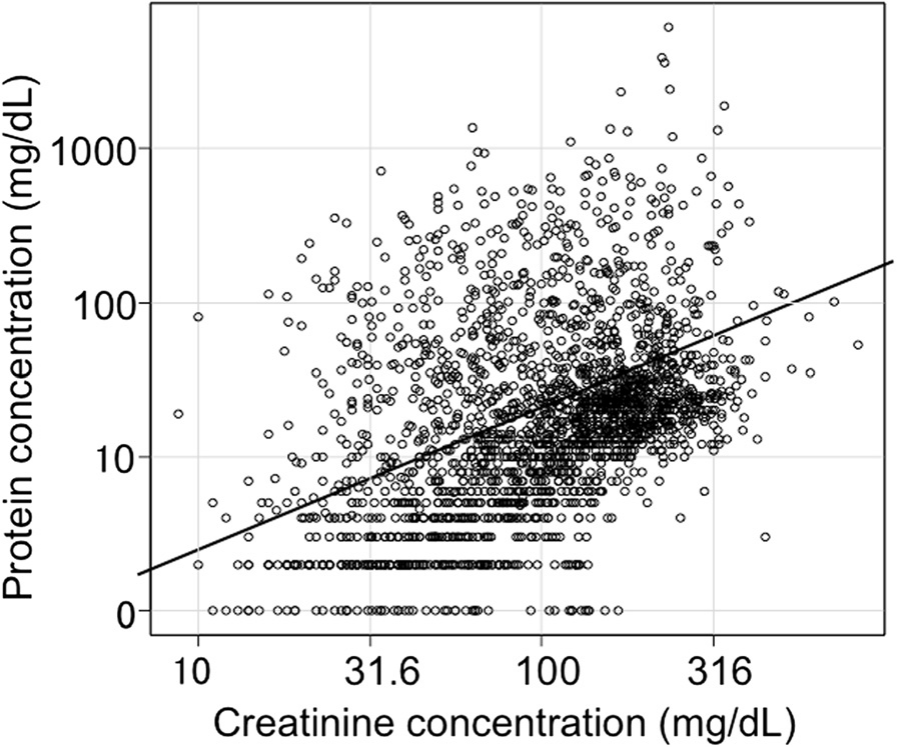
Protein and creatinine concentrations in 2212 urine samples from 1033 pregnant women. The oblique line differentiates the area of P/Cr > 0.27 (upper area) from P/Cr < 0.27 (lower area). In 2212 spot urine samples (1924 antenatal urine samples and 288 postnatal urine samples) from 1033 women, median [Cr] was 105 mg/dL and varied greatly, ranging from 8.1 to 831 mg/dL. Median [P] (range) was 17 (0.0–6170) mg/dL. There were 6 urine samples with [Cr] > 500 mg/dL. Five of the 6 urine samples (from 5 women) with [Cr] > 500 mg/dL were collected at GW 12.7 (8.7–20.3) and the other was at GW 31. The frequency of dilute urine samples with lower [Cr] was significantly higher among postnatal than antenatal urine samples: 4.9 % (14/288) vs. 2.1 % (40/1924), respectively (*P* = 0.012), for [Cr] < 20 mg/dL; and 9.4 % (27/288) vs. 3.8 % (73/1924), respectively (*P* = 0.0001), for [Cr] < 25 mg/dL

In the 2212 urine specimens, median [Cr] was 105 mg and varied greatly ranging from 8.7 to 831 mg/dL (Fig. 1). Median [P] was 17 mg/dL and varied from 0.0 mg/dL to 6170 mg/dL. Concentrated urine samples with [Cr] > 500 mg/dL were collected from women in earlier stages of pregnancy. Diluted urine with lower [Cr] was significantly more common in postnatal urine samples than in those obtained antenatally (see legend for Fig. 1).

Of the 2212 urine samples from 1033 women, 877 (39.6 %), 934 (42.2 %), 252 (11.4 %), and 149 (6.7 %) were negative or equivocal, 1+, 2+, and 3+ on dipstick tests, respectively (Table 2). For prediction of SPIP with dipstick test in this population, false negative test results occurred in 8.8 % (77/877) of samples, and overall false positive test results occurred in 59 % (787/1335) of samples. The false positive rate was 78 % (733/934) for 1+ on dipstick test, 21 % (52/252) for 2+ on dipstick test, and 1.3 % (2/149) for 3+ on dipstick test. It was notable that [Cr] was significantly and consistently higher in urine specimens without SPIP than in those with SPIP at any dipstick test result. In addition, [Cr] was higher for urine specimens with stronger positive dipstick test results.

**Table 2:** Relationship between dipstick test results and P/Cr test results

	P/Cr test		
Dipstick test results	SPIP(%)	[P] (mg/dL)	[Cr] (mg/dL)
Negative/equivocal (*n* = 877)			
SPIP present (*n* = 77)	8.8	18.3 (4.0–323)^a^	45.0 (11.0–194)^a^
SPIP absent (*n* = 800)		5.0 (0.0–50.0)	69.5 (10.0–446)
Positive with 1+ (*n* = 934)			
SPIP present (*n* = 201)	21.5	42.0 (6.0–302)^a^	71.0 (8.7–286)^a^
SPIP absent(*n* = 733)		19.0 (1.0–101)	165 (10.0–831)
Positive with 2+ (*n* = 252)			
SPIP present (*n* = 200)	79.4	100 (25.0–704)^a^	105 (18.0–312)^a^
SPIP absent (*n* = 52)		28.0 (1.0–119)	202 (27.0–598)
Positive with 3+ (*n* = 149)			
SPIP present (*n* = 147)	98.7	331 (12.0–6170)^a^	128 (10.0–401)^a^
SPIP absent (*n* = 2)		79.5 (46.0–113)	383 (259–507)

[P], protein concentration; [Cr], creatinine concentration; SPIP, significant proteinuria in pregnancy defined as P/C > 0.27

^a^, *P* < 0.05 vs. samples without SPIP

**Fig. 2:**
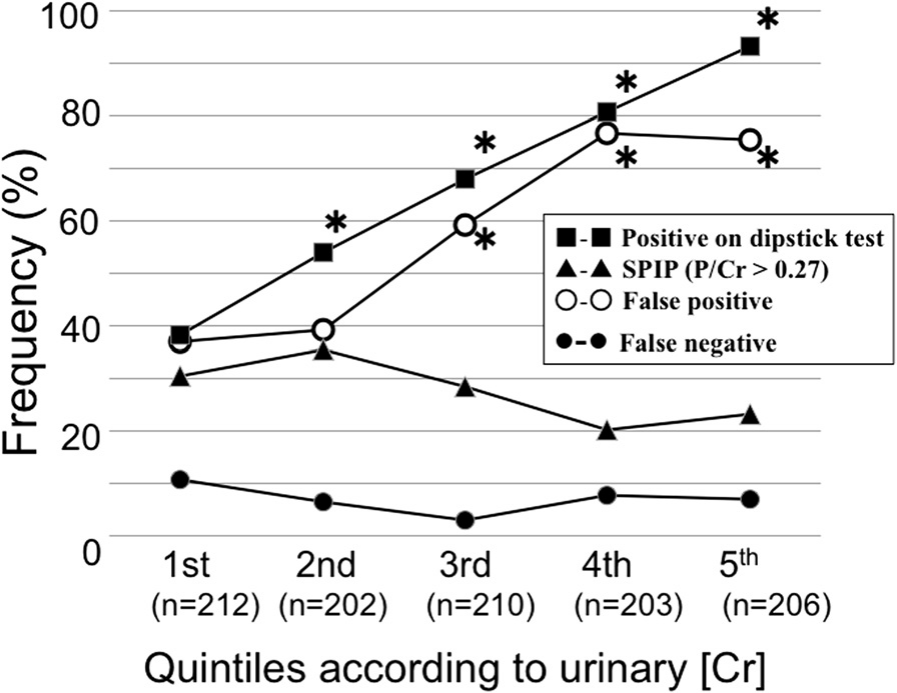
Frequencies of SPIP, and of positive, false positive, and false negative dipstick test results according to urinary creatinine concentration. *, *P* < 0.05 vs. value of the 1st quintile group. This analysis was performed in 1033 urine samples from 1033 women. For women with available antenatal P/Cr test results (*n* = 1004), data of the P/Cr test performed on the day closest to the day of delivery were used. In women with availability of only postnatal P/Cr test results (*n* = 29), data of the P/Cr test performed on the day closest to the day of delivery were used. Urine specimens were grouped into quintiles according to [Cr]: 1st quintile, [Cr] = 10.0–53.9 mg/dL; 2nd quintile, [Cr] = 54.0–90.0 mg/dL; 3rd quintile, [Cr] = 90.1–133 mg/dL; 4th quintile, [Cr] = 133.1–190 mg/dL; and 5th quintile, [Cr] = 190.1–606 mg/dL. False positive rates on dipstick test for prediction of SPIP were 37.0 % (30/81), 39.4 % (43/109), 59.4 % (85/143), 76.8 % (126/164), and 75.5 % (145/192) for the 1st, 2nd, 3rd, 4th, and 5th quintile groups, respectively. False negative rates on dipstick test for prediction of SPIP were 10.7 % (14/131), 6.5 % (6/93), 3.0 % (2/67), 7.7 % (3/39), and 7.1 % (1/14) for the 1st, 2nd, 3rd, 4th, and 5th quintile groups, respectively. The dipstick test gave false positive results exclusively in all urine specimens with [Cr] > 500 mg/dL for prediction of SPIP. The median (range) [P] increased with increasing [Cr]; 5 (1.0–493), 12 (1.0–961), 17 (1.0–1103), 22 (1.0–1342), and 28 (1.0–6170) mg/dL for the 1st, 2nd, 3rd, 4th, and 5th quintile groups, respectively, while corresponding those for women with positive dipstick test results were 27 (1.0–493) (*n* = 81), 27 (1.0–961) (*n* = 109), 23 (1.0–1103) (*n* = 143), 23 (1.0–1342) (*n* = 164), and 30 (1.0–6170) (*n* = 192) mg/dL for the 1st, 2nd, 3rd, 4th, and 5th quintile groups, respectively. Percentage of women with [P] ≥ 30 mg/dL increased with increasing [Cr]; 19 % (40/212), 26 % (53/202), 28 % (59/210), 28 % (57/203), and 49 % (100/208) for the 1st, 2nd, 3rd, 4th, and 5th quintile groups, respectively

As expected, the frequency of positive dipstick test results increased with increasing [Cr], while frequency of SPIP was lower in urine samples with higher [Cr] (Fig. 2), clearly indicating that false positive dipstick test results were likely to occur in concentrated urine samples with higher [Cr] for prediction of SPIP.

Postpartum urine samples were more dilute than those obtained antepartum (Table 3): in the 178 women for whom both ante- and postnatal urine samples were available, [Cr] was significantly lower for postnatal urine samples than for those obtained antenatally. Although not statistically significant, the frequency of SPIP among urine samples with negative/equivocal dipstick test results was higher for postnatal than antenatal urine samples (28 % [25/89] vs. 16 % [7/43], respectively, *P* = 0.1933), suggesting that false negative dipstick test results were more likely to occur postpartum than antepartum.

**Table 3:** Comparison between antenatal and postnatal urine samples

	Antenatal urine	Postnatal urine
No. of specimens	178	178
GW at sampling	36.1 (10–41)	NA
Days before delivery	2.5 (0–195)	NA
Postpartum days at sampling	NA	5.0 (0–63)
[P] (mg/dL)	86 (0.0–6170)	25.5 (1.0–648)^a^
[Cr] (mg/dL)	100 (10–401)	60 (8.7–297)^a^
Negative/equivocal dipstick test result		
No. of specimens	43	89
[P] (mg/dL)	8.0 (0.0–56)	10.0 (1.0–193)
[Cr] (mg/dL)	68.0 (10.0–310)	55.0 (14.0–297)
P/Cr	0.09 (0.0–0.71)	0.17 (0.01–3.51)
SPIP (P/Cr > 0.27)	7 (16.3 %)	25 (28.1 %)

178 paired urine sample from 178 women were analyzed. GW, gestational week; NA, not applicable; [P], protein concentration; [Cr], creatinine concentration; and SPIP, significant proteinuria in pregnancy

^a^, *P*  <  0.05 vs. antenatal samples

Finally, the associations between reasons for the first P/Cr test and P/Cr test results were examined (Table 4). Reasons for the first P/Cr test were specified in 1028 (99.5 %) of the 1033 women enrolled in the study. At the first P/Cr test, hypertension was already confirmed in 222 (22 %) but not in 806 (78 %) of these 1028 women. Among women with similar dipstick test results, the risk of having SPIP was consistently and significantly higher for hypertensive than for normotensive women: 18 % (14/77) vs. 3.2 % (8/251) for negative/equivocal dipstick test result, 47 % (26/55) vs. 8.7 % (37/425) for 1+ dipstick test result, and 91 % (82/90) vs. 59 % (44/75) for ≥ 2+ dipstick test result. The risk of SPIP was 16 % (9/55) for normotensive women with 1+ dipstick test result on two successive antenatal care visits. It was notable that [Cr] in the urine samples with ≥ 1+ on dipstick test was significantly lower for hypertensive than normotensive women.

**Table 4:** Association between reasons for first P/Cr test and P/Cr test results

	P/Cr test result		
Reason for the first time P/Cr test	[P]	[Cr]	SPIP (P/Cr > 0.27)
After confirmation of hypertension onset (*n* = 222) with following dipstick test results:			
Negative/equivocal (*n* = 77)	9 (1–323)^a^	66 (11–194)	14 (18 %)^a^
Positive with 1+ (*n* = 55)	25 (5–314)^a^	117(20–402)^a^	26 (47 %)^a^
Positive with ≥ 2+ (*n* = 90)	185 (6–3890)^a^	133 (18–533)^a^	82 (91 %)^a^
No hypertension onset (*n* = 806) with following dipstick test results:			
Negative/equivocal (*n* = 251)	4 (0–60)	63 (11–446)	8 (3.2 %)
Positive with 1+ (*n* = 425)	19 (1–196)	162 (10–708)	37 (8.7 %)
Positive with ≥ 2+ (*n* = 75)	71 (1–866)	191 (27–598)	44 (59 %)
Positive with 1+ at two successive visits (*n* = 55)	21 (1–192)	146 (26–364)	9 (16 %)

The reason for the first P/Cr test was specified in 1028 of the 1033 women

[P], protein concentration; [Cr], creatinine concentration; and SPIP, significant proteinuria in pregnancy

^a^, *P* < 0.05 vs. corresponding value for the group without hypertension

## Discussion

In this study population in which approximately 20, 7, and 12 % of women developed PE, gestational hypertension, and isolated SPIP, respectively, the following three points were emphasized: (1) dipstick test results were closely related to [Cr], and false positive results were likely to occur in concentrated urine samples with higher [Cr]; (2) postpartum urine samples had significantly lower [Cr] compared to antepartum urine samples; and (3) in comparison between normotensive and hypertensive women with similar dipstick test result, the risk of having SPIP was consistently higher for hypertensive than normotensive women at any dipstick test result. For example, among women with a result of 1+ on dipstick test, SPIP was present in 47 vs. 8.7 % of hypertensive vs. normotensive women, respectively. Even among women with negative or equivocal dipstick test results, SPIP was present in as many as 18 % of hypertensive women, while this rate was 3.2 % in normotensive women.

PE is a life-threatening complication [1] and hospitalized care is currently recommended for women diagnosed with this condition [17, 19]. As the dipstick test is widely used for screening of SPIP, it is very important to determine which women should undergo confirmation tests, such as the P/Cr test, for diagnosis of SPIP. However, the frequency of false positive results on dipstick test is high [8–10], as confirmed in the present study. There have been only a few reports focusing on the association between the results of dipstick and P/Cr tests [9, 10]. Therefore, better characterization of the screening characteristics of the dipstick test according to various clinical situations would help clinicians in prediction of SPIP with this test.

This study confirmed that urine of pregnant women had varied [Cr], ranging from 8.1 to 862 mg/dL (median, 105 mg/dL), suggesting that the kidneys are able to concentrate urine by approximately 100-fold in pregnancy. In addition, it was clearly demonstrated that the false positive rate on the dipstick test increased with increasing [Cr] for prediction of SPIP (Fig. 2). All of six urine samples with very high [Cr] > 500 mg/dL exhibited exclusively false positive results on dipstick test (see legends for Figs. 1 and 2). Five of these six urine samples were collected at earlier stages of pregnancy. It was speculated that these women with extremely high [Cr] suffered from hyperemesis and dehydration, and excreted concentrated urine samples. This scenario explained our clinical impression that a positive dipstick test result was relatively common in women in early stages of pregnancy. In this context, it may be important to note that postpartum urine samples were more dilute than those obtained antepartum (Table 3). A false negative dipstick test result was relatively common (approximately 11 %) in dilute urine samples with [Cr] < 54 mg/dL (Fig. 2). The more dilute postpartum urine may be explained as follows. Water retention occurs physiologically in normal pregnancy. The process involved in the retention of water during pregnancy is reversed by parturition, and the excess water in the interstitial space returns into the intravascular space, resulting in a fall in hematocrit value [20] and the excess water is then excreted as urine with lower [Cr] postpartum.

In this study, the risk of having SPIP among women with similar results on dipstick test was significantly higher for hypertensive than normotensive women at any dipstick test result, including negative/equivocal test results (Table 4). To our knowledge, this is the first report of this phenomenon, and it suggested that a false positive dipstick test result was less likely in urine samples of hypertensive women. As shown clearly in this study, the dipstick test results were associated with [Cr] and the false positive rate was lower in urine samples with lower [Cr]. Indeed, urine samples of hypertensive women were less concentrated than those of normotensive women (Table 4), suggesting that hypertension may affect renal function with respect to urine concentration. In addition to the mechanism underlying this phenomenon, it may be clinically important that women after confirmation of hypertension had significantly higher risk of SPIP at any dipstick test result compared to those who remained normotensive. In this population, the risks of SPIP were 18, 47, and 91 % for hypertensive women with negative/equivocal, 1+, and ≥ 2+ results on dipstick test, respectively. Corresponding values were 3.2, 8.7, and 59 % for normotensive women, respectively. These observations suggested that hypertensive women with dipstick test result ≥ 1+ as well as normotensive women with dipstick test result ≥ 2+ should be recommended to undergo a confirmation test for SPIP.

In an earlier single-center study in pregnant women with GW 30–36 who did not develop PE, as many as 28 % of women exhibited a 1+ result on dipstick test at least once, and 5.9 % of women exhibited 1+ on dipstick test at two successive antenatal care visits [10]. In this study, SPIP was confirmed in 8.7 % (37/425) of normotensive women with a result of 1 + on dipstick test. This SPIP prevalence rate increased to 16 % (9/55) in women with a result of 1 + on dipstick test at two successive antenatal care visits, which was somewhat lower than the value of 30 % reported previously [10]. However, based on these results, women with a dipstick test result of 1 + at two successive antenatal care visits appeared to have considerably higher risk of SPIP compared to those with a dipstick test result of 1 + only once. These women were also considered as candidates for confirmation test for SPIP.

## Conclusion

Although the dipstick test may be appropriate for screening of SPIP on the basis of both cost and rapidity, the dipstick test was likely to show a false positive test result with increasing [Cr]. However, women with confirmed hypertension were less likely to exhibit a false positive test result on dipstick test compared to those who remained normotensive. The risk of SPIP was 74 % (108/145) in hypertensive women with urine samples showing a dipstick test result ≥ 1+ (Table 4). Even in normotensive women, the risk of SPIP was more than 50 % when their urine samples exhibited dipstick test results ≥ 2+. These results suggested that hypertensive women with dipstick test results ≥ 1+ as well as normotensive women with dipstick test results ≥ 2+ are candidates for confirmation test for SPIP. In addition, postpartum urine samples were more dilute than antepartum urine samples. It should be noted that false negative results are relatively common in such urine samples with lower [Cr].
